# Gold nanorods with iron oxide dual-modal bioprobes in SERS-MRI enable accurate programmed cell death ligand-1 expression detection in triple-negative breast cancer

**DOI:** 10.1063/5.0152846

**Published:** 2023-05-23

**Authors:** Ting Pan, Dinghu Zhang, Xiaoxia Wu, Zihou Li, Hui Zeng, Xiawei Xu, Chenguang Zhang, Yiwei He, Yuanchuan Gong, Pin Wang, Quanliang Mao, Junlie Yao, Jie Lin, Aiguo Wu, Guoliang Shao

**Affiliations:** 1 Department of Imaging and Nuclear Medicine, Wannan Medical College, Wenchang Xi Road No.22, Wuhu 241002, People's Republic of China; 2 Department of Interventional Radiology, Zhejiang Cancer Hospital, Institute of Basic Medicine and Cancer (IBMC), Chinese Academy of Sciences, Hangzhou, Zhejiang 310022, People's Republic of China; 3 Cixi Institute of Biomedical Engineering, International Cooperation Base of Biomedical Materials Technology and Application, Chinese Academy of Science (CAS) Key Laboratory of Magnetic Materials and Devices and Zhejiang Engineering Research Center for Biomedical Materials, Ningbo Institute of Materials Technology and Engineering, CAS, Ningbo 315201, People's Republic of China; 4 Advanced Energy Science and Technology Guangdong Laboratory, Huizhou 516000, People's Republic of China

## Abstract

The efficiency of immunotherapy for triple-negative breast cancer (TNBC) is relatively low due to the difficulty in accurately detecting immune checkpoints. The detection of TNBC-related programmed cell death ligand-1 (PD-L1) expression is important to guide immunotherapy and improve treatment efficiency. Surface-enhanced Raman spectroscopy (SERS) and magnetic resonance (MR) imaging exhibit great potential for early TNBC diagnosis. SERS, an optical imaging mode, has the advantages of high detection sensitivity, good spatial resolution, and “fingerprint” spectral characteristics; however, the shallow detection penetration of SERS bioprobes limits its application *in vivo*. MR has the advantages of allowing deep penetration with no radiation; however, its spatial resolution needs to be improved. SERS and MR have complementary imaging features for tumor marker detection. In this study, gold nanorod and ultrasmall iron oxide nanoparticle composites were developed as dual-modal bioprobes for SERS-MRI to detect PD-L1 expression. Anti-PD-L1 (aPD-L1) was utilized to improve the targeting ability and specificity of PD-L1 expression detection. TNBC cells expressing PD-L1 were accurately detected via the SERS imaging mode *in vitro*, which can image at the single-cell level. In addition, bioprobe accumulation in PD-L1 expression-related tumor-bearing mice was simply and dynamically monitored and analyzed *in vivo* using MR and SERS. To the best of our knowledge, this is the first time a SERS-MRI dual-modal bioprobe combined with a PD-L1 antibody has been successfully used to detect PD-L1 expression in TNBC. This work paves the way for the design of high-performance bioprobe-based contrast agents for the clinical immunotherapy of TNBC.

## INTRODUCTION

The triple-negative breast cancer (TNBC) is one of most aggressive forms and has a very poor prognosis.^1,2^ Nonetheless, the treatment for TNBC has some promising outcomes attributed to the programmed cell death protein 1 (PD-1) and programmed cell death ligand-1 (PD-L1) immune checkpoint inhibitors (ICI).^3^ The detection and analysis of PD-1/PD-L1 checkpoints could help direct individual treatment strategies, which could greatly affect the efficacy and responsiveness of ICI.^4^ The conventional methods for immune checkpoint detection include immunohistochemistry (IHC), western blot (WB), enzyme linked immunosorbent assay (ELISA), and so on.^5^ However, all of aforementioned technologies require tissue sample collection, which can be time consuming while introducing pain and risk to patients. It is most important to develop new noninvasive, dynamic, and quantitative technologies for whole-body imaging to map the spatiotemporal heterogeneity of PD-L1 expression.^6^ Positron emission tomography (PET), a noninvasive and high-sensitive imaging method, plays a vital role in the management of ICI therapy by using different nuclide molecular probes and metabolic parameters in preclinical studies and helps diagnose and treat disease in clinics.^7^ Bensch *et al.* reported the feasibility of zirconium-89-labeled anti-PD-L1 for PD-L1 imaging using PET imaging for the first time in the year of 2018.^8^ However, the zirconium-89 labeled antibody showed a long biological half-life *in vivo*, which would cause damage via exposure to radionuclide probes and x-ray emission sources.^6^ Therefore, it is necessary to develop safe and noninvasive molecular imaging methods to monitor PD-1/PD-L1 checkpoint expression before, during, and after immunotherapy.

Magnetic resonance (MR) imaging, a nonradiative imaging technology, can offer abundant physiological and anatomic information about tumor tissue and is widely utilized for cancer diagnosis. Although gadolinium-based agents are common *T*_1_-enhanced contrast agents for clinical MR imaging (MRI) application, serious nephrotoxicity side effects and allergic reactions can occur.^9^ Thus, iron oxide nanoparticle (IO NP)-based *T*_2_-enhanced contrast agents with better biocompatibility have attracted increasing attention in recent years. It is noteworthy that ultrasmall IO NPs, with a size distribution of 1–10 nm, have both *T*_1_- and *T*_2_-enhanced capabilities.^10–13^ IO NP-based MRI bioprobes have the advantage of deep penetration, but their limited detection sensitivity restricts their application with low-concentration biomarkers. Surface-enhanced Raman scattering (SERS) technology is based on the molecular and lattice vibration of light inelastic scattering.^14–16^ SERS spectra is regarded as a promising molecular imaging method due to its significant advantages including high detection sensitivity, non-destructiveness, fast response time, and unique information supply.^17–19^ For SERS substrates, noble metal materials play a vital role in boosting target molecular Raman signal owing to the surface plasmon resonance (SPR) effect.^20,21^ Although silver NPs produce the largest SERS enhancement factor,^17^ a certain amount biotoxicity limits silver SERS substrate application in the field of biological detection. Gold NPs, another high-performance SERS signal amplifier, have been reported to have important applications in the biosensing field, such as circulating tumor cell detection, tumor cell bioimaging, and tumor cell differentiation.^22–24^ In particular, gold nanorods (GRs) have been reported as good SERS bioprobes in biosensing applications because of their near-infrared absorption wavelength, which is determined by the nanorod morphology-produced large longitudinal SPR effect.^25–27^ GR-based SERS bioprobes exhibit great application potential in tumor PD-L1 expression detection *in vitro*. However, the relatively shallow penetration of SERS technology is an inevitable weakness of *in vivo* biodetection and bioimaging.

**Scheme 1.**
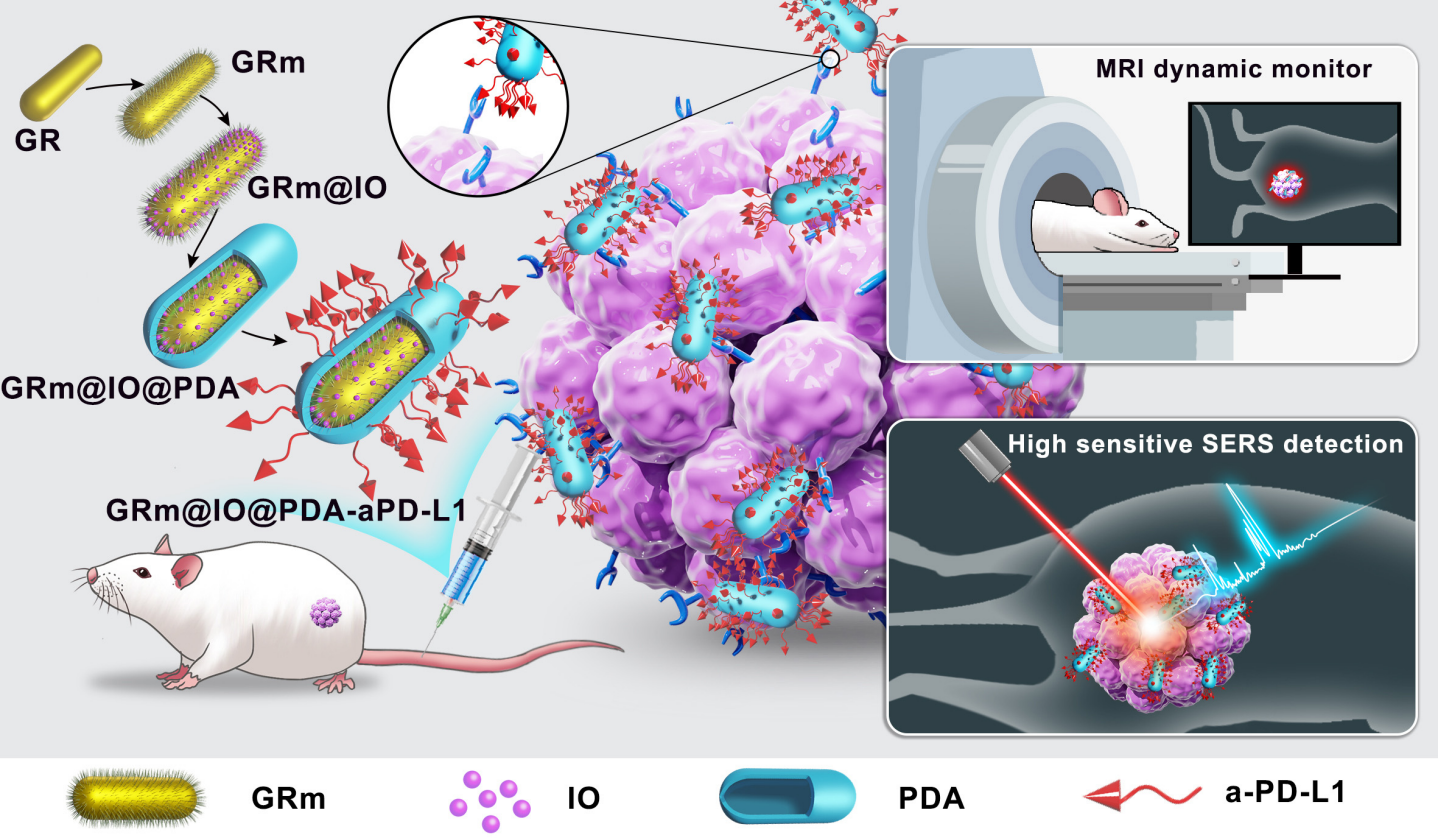
Design of the programmed cell death ligand-1 (PD-L1) targeted SERS-MRI dual-modal bioprobes (GRm@IO@PDA-aPD-L1) for specific and sensitive detection of PD-L1 expression in tumor.

SERS and MRI modalities can complement tumor diagnostic modes and offer complementary bioimaging features with high sensitivity and deep penetration. The development of a SERS–MRI double-mode imaging bioprobe could be a promising method for accurately detecting TNBC-related PD-L1 expression. In this study, GRs with ultrasmall iron oxide (IO) composite bioprobes (GR@IO) were designed to integrate SERS and MR imaging properties, as detailed in Scheme 1. 4-mercaptobenzoic acid (4MBA) molecules were adsorbed on the synthesized GR@IO SERS samples (GRm@IO); the uniform SERS substrate morphology was beneficial for acquiring highly reproducible SERS signals. The GRm@IO composite was modified with aPD-L1 to improve the detection specificity and targeting ability for tumor PD-L1 expression detection. Almost no toxicity was observed for the GRm@IO composite SERS bioprobes based on a Cell Counting Kit-8 (CCK-8) assay. High PD-L1 expression in single TNBC cells, compared to that of MCF-7 cells, was accurately detected by the SERS detection/imaging mode using the GRm@IO composite bioprobes *in vitro* with detection sensitivity at the single-cell level. Moreover, PD-L1 expression in positive and negative cancer cells was accurately distinguished through the MR mode of GRm@IO composite bioprobes, and dynamic monitoring of bioprobe aggregation in tumor-bearing mice was realized *in vivo* via MR. To the best of our knowledge, this is the first time a GR@IO-based bioprobe combined with a PD-L1 antibody has been developed to qualitatively distinguish PD-L1 expression in breast cancer cells based on SERS-MRI dual-modal bioprobes *in vitro* and *in vivo*. This study will pave the way for the design of high-performance SERS-MRI bioprobes for tumor PD-L1 expression detection, guide TNBC clinical immunotherapy, and achieve tumor precision medicine.

## RESULTS AND DISCUSSION

### Synthesis and characterization of aPD-L1-conjugated GRm@IO

**FIG. 1.**
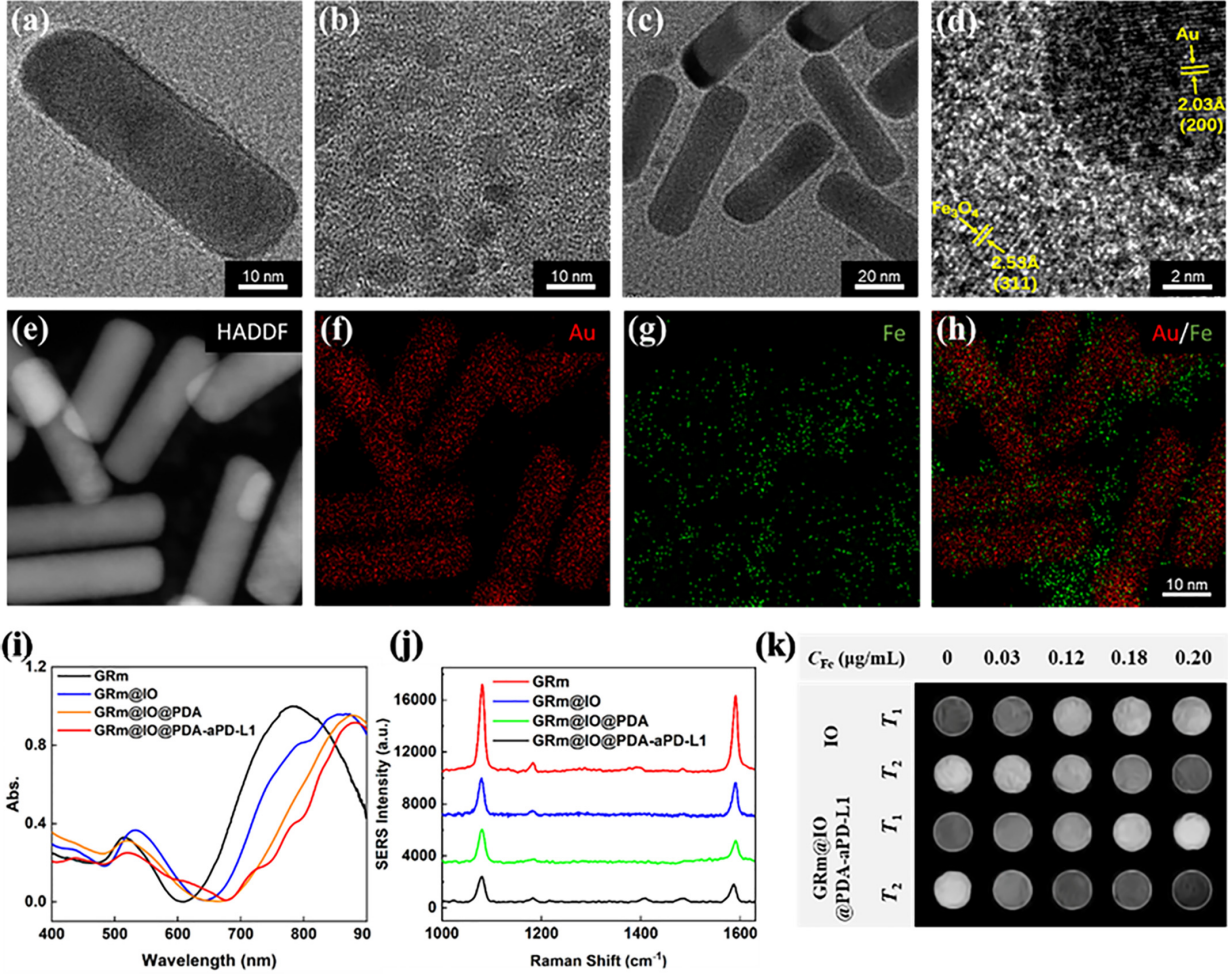
Transmission electron microscopy (TEM) images of (a) GR, (b) IO, (c) GRm@IO, and (d) high-resolution TEM (HRTEM) images of GRm@IO (the crystal lattice of GRm and IO, respectively). (e)–(h) HADDF and elemental mapping for the gold and iron in GRm@IO. (i) and (j) UV-Vis and Raman spectra of GRm, GRm@IO, GRm@IO@PDA, and GRm@IO@PDA-aPD-L1. Raman test condition for SERS spectra: laser wavelength: 785 nm; laser power: 26.7 mW, 25%; lens: 50 × objective. (k) *T*_1_-weighted and *T*_2_-weighted images of IO and GRm@IO@PDA-aPD-L1 with different Fe concentration (*C*_Fe_: 0–0.2 *μ*g/ml) under the 0.5 T magnetic field (*T*_1_: TR = 500, TE = 18.2 ms; and *T*_2_: TR = 2000, TE = 40 ms).

The design route for the GRm@IO based-bioprobe, which was synthesized via seed growth and nanoprecipitation, is shown in Scheme 1. GRs were prepared using the seed generation method, as previously described,^28^ and 4MBA molecules were absorbed on the surface of the GRs for SERS detecting/imaging. To obtain a substrate with high SERS activity, GRs with a length-to-diameter ratio of three were successfully synthesized by regulating the sodium hydroxide (NaOH) concentration [22.5 *μ*l, 27.5 *μ*l, 32.5 *μ*l (1 mol/l)]. Broad view transmission electron microscopy (TEM) images of the GR samples are shown in Fig. S1(a). UV-visible absorption spectra and SERS spectra results show that 4MBA molecules adsorbed on GRs (GRm), with the length-to-diameter ratio of 3.72 (GRs-3.72) exhibiting the best SERS enhancement under 785 nm laser illumination [Fig. S1(b) and S1(c)]. The high SERS activity of the GRs-3.72 sample can be explained by the fact that its absorption peak is closer to 785 nm than those of the other two samples. The TEM image of a single GRs-3.72 sample is shown in Fig. 1(a). The length was ∼60 nm, the diameter was ∼16 nm, and the size of the synthesized IO NPs was ∼6 nm [Fig. 1(b)]. The zeta potentials of GRm and IO NPs were positive and negative, respectively, as shown in Fig. S2. This was attributed to the modification of SH-PEG-NH_2_ in the GRs and polyacrylic acid (PAA) on the surfaces of the IO NPs. GRm@IO was prepared based on the amidation reaction between the –COOH of PAA and the -NH_2_ of SH-PEG-NH_2_,^29^ as observed in the TEM image [Fig. 1(c)]. The high-resolution transmission electron microscopy image (HRTEM) in Fig. 1(d) shows that the adjacent lattice fringes of the GR and IO NPs were 0.203 and 0.253 nm, which correspond to the (200) plane lattice constant of Au,^30^ and the (311) plane lattice constant of IO,^31^ respectively. The high-angle annular dark-field STEM (HAADF-STEM) results of the Au/Fe elemental images further confirm that GRm@IO composites were successfully obtained [Figs. 1(e)–1(h)].

To increase the biocompatibility of the GRm@IO composite in solution, a layer of polydopamine (PDA) was wrapped on the surface of the material. aPD-L1 was modified with GRm@IO@PDA to endow the GRm@IO-based SERS bioprobe with high targeting efficiency to PD-L1 expression-related tumor sites. The PDA layer on the GRm@IO composite was observed by TEM (Fig. S3) and N/Au/Fe elemental mapping images were observed in the HAADF-STEM images shown in Fig. S4, which indicated the presence of a PDA layer on the SERS bioprobe. In addition, UV-vis absorption spectra [Fig. 1(i)] and dynamic light scattering (Fig. S5) further verified the presence of the dopamine layer and aPD-L1 antibodies. As the GRm@IO composite was modified with the dopamine layer and aPD-L1 antibodies, the absorption spectra were shifted apparently toward red, and the particle sizes of hydration gradually increased. aPD-L1 antibodies modified on dopamine layer through the Schiff base reaction was affirmed by the zeta potential results (Fig. S2).^32^ Electrical properties of the bioprobes became positive as the hydroxyl group on dopamine was consumed in the amido bonding formation process.^33^ In addition, green fluorescence images of Alexa Fluor 488-labeled aPD-L1 were observed on the GRm@IO-based SERS bioprobes (Fig. S6), demonstrating that aPD-L1 antibodies were effectively linked to the GRm@IO@PDA surface, and GRm@IO@PDA-aPD-L1 SERS bioprobes were successfully synthesized. Importantly, although PDA and aPD-L1 were modified on the GRm@IO surface, the 4MBA SERS signals were clearly visible, and the signal-to-noise ratio of the designed SERS bioprobes was sufficient for tumor PD-L1 expression detection and imaging [Fig. 1(j)]. The MR contrast agent performance of the GRm@IO-based bioprobes was evaluated using MR equipment (0.5 T). *T*_1_- and *T*_2_-weighted images of IO and GRm@IO@PDA-aPD-L1 with different Fe concentrations (0–0.2 *μ*g/ml) are shown in Fig. 1(k). Enhanced GRm@IO@PDA-aPD-L1 nanoparticle abilities were observed in *T*_1_ and *T*_2_ MR images, indicating they could be used as bimodal MR contrast agents in tumor imaging.^9^ Therefore, these bioprobes exhibit SERS-MRI dual-modal detection/imaging capabilities for targeted tumors with PD-L1 expression.

**FIG. 2.**
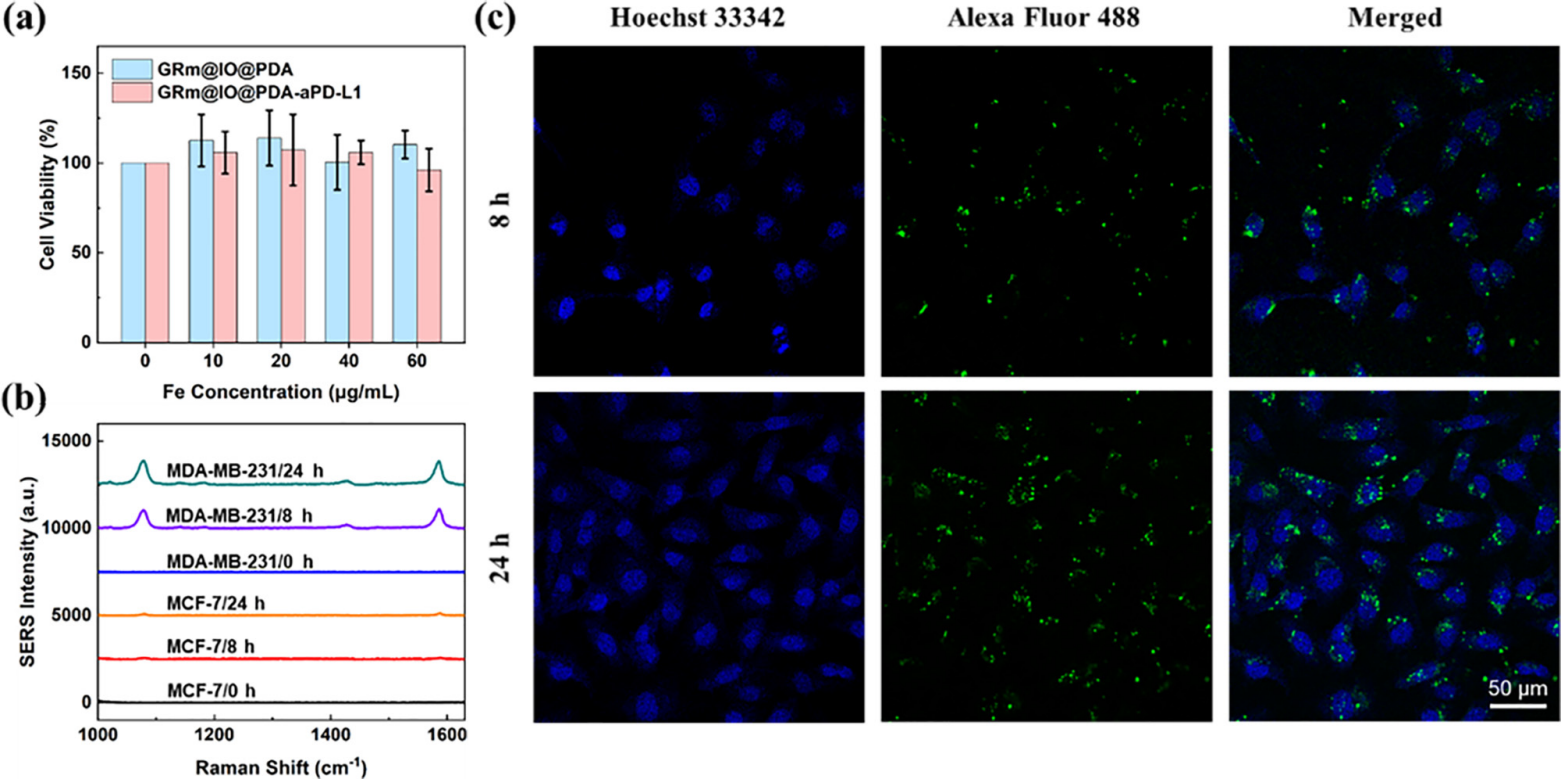
(a) Cell viability of MDA-MB-231 cells incubated with GRm@IO@PDA-aPD-L1 bioprobes at different Fe concentration. (b) SERS spectra of MDA-MB-231 and MCF-7 cells incubated with GRm@IO@PDA-aPD-L1 bioprobes for different incubated time. Raman test condition for SERS spectra: laser wavelength: 785 nm; laser power: 26.7 mW, 25%; lens: 50 × objective. (c) Confocal laser scanning microscopy (CLSM) images of MDA-MB-231 cells with Alexa Fluor 488 marked bioprobes (GRm@IO@PDA-aPD-L1-488) for different incubation time. The cells were treated with Hoechst 33342 (Em: 430–490 nm; and Ex: 405 nm), and GRm@IO@PDA-aPD-L1-488 were excited at the wavelength of 488 nm, and the fluorescence images at emission wavelengths are at 500–550 nm.

To assess the capability of detecting PD-L1 expression with our SERS-MRI dual-modal bioprobes, human breast cancer cell lines of MDA-MB-231 and MCF-7, which have different PD-L1 expression levels, were selected as the research model.^1^ First, biocompatibility was evaluated to ensure safety. The cytotoxicity of the GRm@IO-based bioprobes in MDA-MB-231 and MCF-7 cancer cells was studied using the CCK-8 assay [Figs. 2(a) and S7]. The CCK-8 assay results illustrate that the GRm@IO@PDA@aPD-L1 bioprobe displays almost no cytotoxicity, and its good biocompatibility supports the subsequent detection of PD-L1 expression in tumor cells. The SERS detection mode of the GRm@IO@PDA-aPD-L1 bioprobe was used to differentiate between MDA-MB-231 and MCF-7 cancer cells with different PD-L1 expression levels [Fig. 2(b)]. We found that MDA-MB-231 tumor cells presented higher SERS signal intensities than MCF-7 tumor cells, and it seemed that the SERS signal intensity of MDA-MB-231 cells incubated with 24 h was higher than that incubation time of 8 h due to longer uptake time. SERS intensities strongly depended on the number of GRm@IO-based bioprobes in tumor cells, which was determined by PD-L1 expression in cancer cells. According to the SERS results, MDA-MB-231 tumor cells showed higher PD-L1 expression than MCF-7 cancer cells. To confirm these results, immunofluorescence measurements of the targeting ability of the GRm@IO@PDA-aPD-L1 bioprobe for MDA-MB-231 and MCF-7 tumor cells with different co-incubation times were performed [Figs. 2(c) and S8). Fluorescence images confirmed that more bioprobes were present in MDA-MB-231 tumor cells, which may have been determined by differences in tumor PD-L1 expression. Flow cytometry results (Fig. S9) further verified that MDA-MB-231 cells had higher PD-L1 expression, resulting in a higher uptake of bioprobes by tumor cells.

### SERS and MRI imaging of the developed imaging probes

**FIG. 3.**
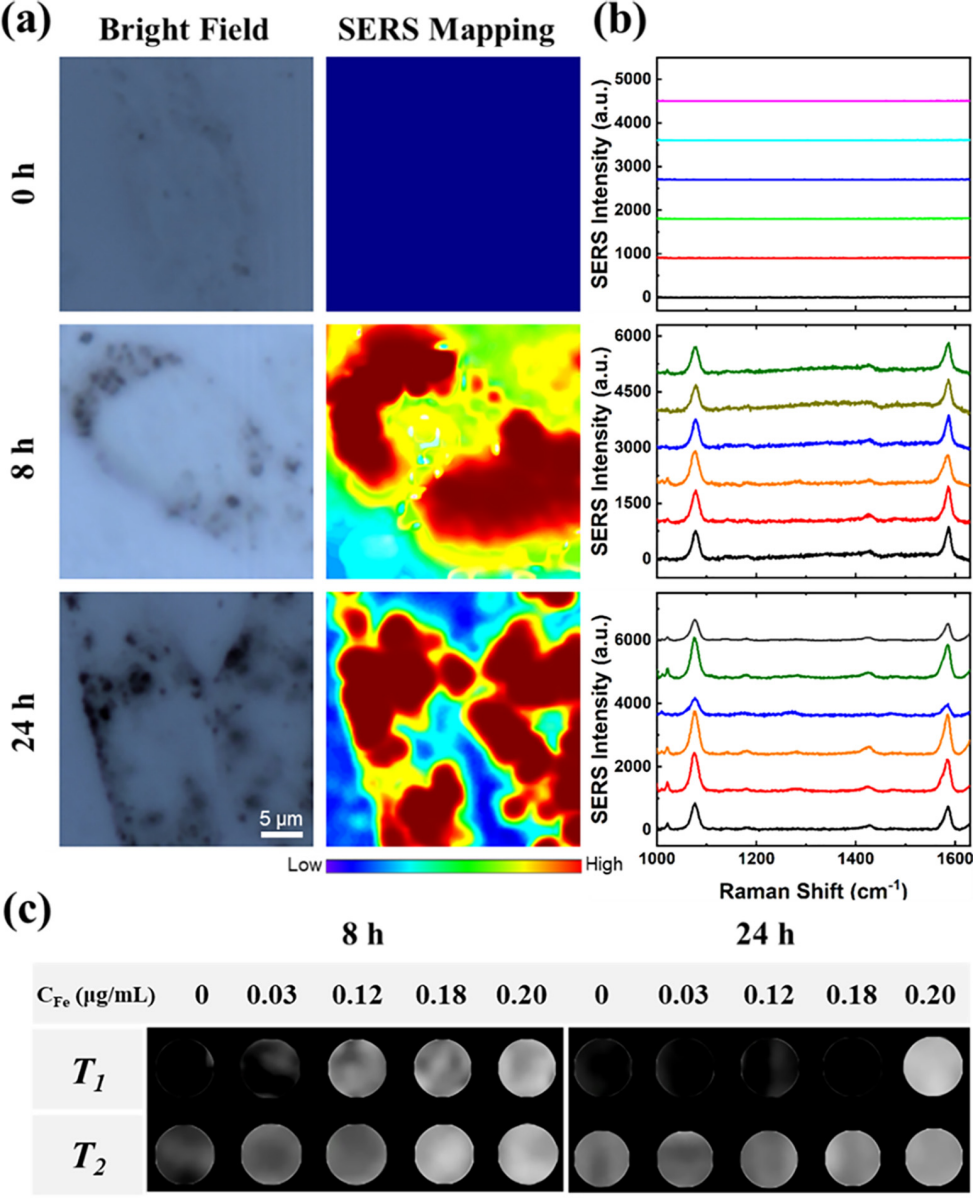
(a) Bright field and SERS mapping images of MDA-MB-231 cells incubated with GRm@IO@PDA-aPD-L1 bioprobes for 8 and 24 h. (b) SERS spectra of random six points from the left MDA-MB-231 cells. Raman test condition for SERS spectra: laser wavelength: 785 nm; laser power: 26.7 mW, 25%; lens: 50 × objective. (c) *T*_1_-weighted and *T*_2_-weighted images of MDA-MB-231 cells incubated with GRm@IO@PDA-aPD-L1 at different Fe concentration (*C*_Fe_: 0–0.2 *μ*g/ml) for 8 and 24 h under 0.5 T magnetic field (*T*_1_: TR = 500, TE = 18.2; and *T*_2_: TR = 2000, TE = 40 ms).

Visual imaging modes based on GRm@IO@PDA-aPD-L1 bioprobes will play a vital role in accurately evaluating average PD-L1 expression in TNBC cells. SERS images of the GRm@IO-based bioprobes co-incubated with MDA-MB-231 and MCF-7 cells for 0, 8, and 24 h are shown in Figs. 3(a) and S10(a), respectively. SERS imaging displayed the details of bioprobe distribution in single cancer cells. The number of SERS bioprobes taken up by TNBC cells was significantly higher than those taken up by MCF-7 cells, which may have been caused by the higher PD-L1 expression in TNBC cells. Profiting from the superiority of single-cell imaging sensitivity, SERS laser spots randomly acquired in single MDA-MB-231 cell processing had higher Raman intensities than those of MCF-7 cells and were clearly contrasted [Fig. 3(b) and S10(b)]. As the co-incubation time increased, the average SERS mapping intensities of MDA-MB-231 cells increased owing to the accumulation of bioprobes in the cells. MRI images of the GRm@IO-based bioprobes in MDA-MB-231 and MCF-7 cells are shown in Figs. 3(c) and S10(c), respectively. GRm@IO@PDA-aPD-L1 groups showed more obvious *T_1_* and *T_2_* signals in TNBC cells than that for MCF-7 cells due to the high uptake and PD-L1 expression level in MDA-MB-231 cells. This is consistent with the results shown in Figs. 2(b), 2(c), and 3(a). The *T*_1_ MR images of MDA-MB-231 cells incubated with GRm@IO@PDA-aPD-L1 for 8 h were brighter than those incubated for 24 h, which may due to the degradation of IO over time in these cells.^34^ The aforementioned SERS mapping and MRI imaging results demonstrate that GRm@IO@PDA-aPD-L1 bioprobes can efficiently target TNBC cells, and that TNBC PD-L1 expression can be accurately detected by these SERS-MRI dual-modal bioprobes.

### *In vivo* SERS-MRI dual-modal imaging of PD-L1 expression

To further evaluate the potential of our biprobes for PD-L1 expression detection in tumors, the *in vivo* biocompatibility of GRm@IO@PDA-aPD-L1 was investigated in Institute of Cancer Research (ICR) mice. Fifteen days after tail vein injection of PBS or GRm@IO@PDA-aPD-L1, the blood and major organs of the mice were collected for hematology analysis and hematoxylin-eosin (H&E) staining to study organ pathology (Figs. S11 and S12). GRm@IO@PDA-aPD-L1 administration had no influence on all indices examined in routine blood tests.^35^ Furthermore, there were no appreciable pathological lesions in the heart, liver, spleen, lung, or kidney of mice treated with GRm@IO@PDA-aPD-L1. This indicated that our bioprobes possessed good biocompatibility for application in PD-L1 expression detection *in vivo*.

**FIG. 4.**
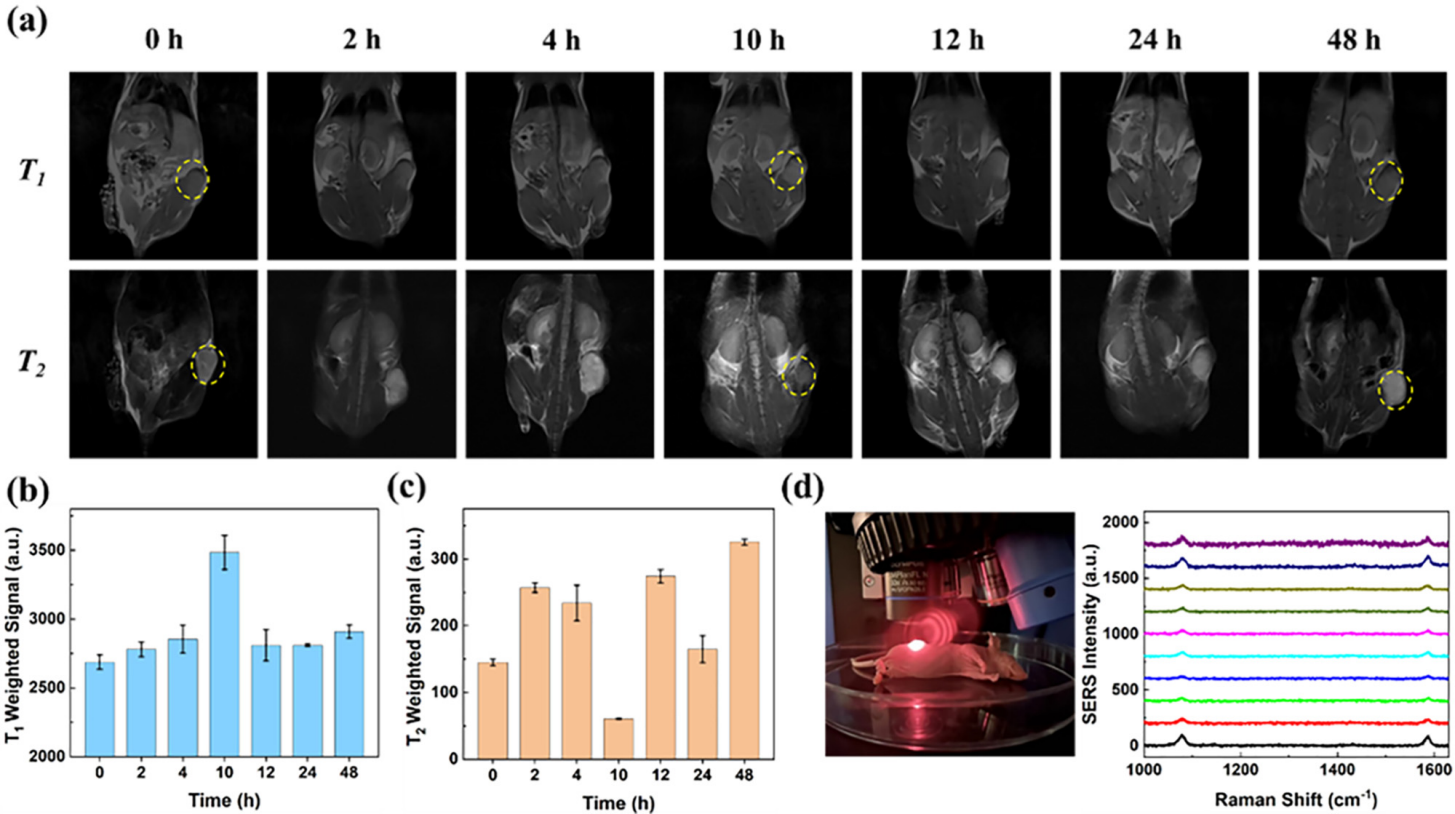
(a) *T*_1_-weighted and *T*_2_-weighted images of MDA-MB-231 cells bearing mice at different time points under the 3.0 T magnetic field. (b) and (c) Relative intensities of *T*_1_ and *T*_2_ signal of tumor bearing mice. (d) SERS spectra of 10 random points from MDA-MB-231 tumor in BALB/c female mouse. Raman test condition for SERS spectra: laser wavelength: 785 nm; laser power: 26.7 mW, 25%; lens: 50 × objective.

GRm@IO-based bioprobes were injected into tumor-bearing mice via the tail vein for *in vivo* PD-L1 expression detection by SERS-MRI. To explore the optimum time for maximum bioprobe accumulation, the amount of bioprobes accumulated in TNBC tumor-bearing mice based on PD-L1 expression was directly and dynamically monitored using MRI, as shown in Fig. 4(a). MR revealed that the optimum time for maximum GRm@IO@PDA-aPD-L1 bioprobe accumulation at TNBC tumor sites was 10 h. This is when the MRI signal was strongest in *T*_1_- and *T*_2_-weighted imaging [Figs. 4(b) and 4(c)]. The relative intensities of *T*_1_- and *T*_2_-weighted signals measured by MRI at different time points [Figs. 4(b) and 4(c)] further confirmed the greater accumulation of GRm@IO@PDA-aPD-L1 bioprobes in tumor tissues. In order to validate the detection specificity of GRm@IO@PDA-aPD-L1 bioprobes, it could be found that there is no obvious cumulative effect of the GRm@IO@PDA bioprobes (200 *μ*l, 0.98 mg/ml) in tumor during 24 h post injection (Fig. S13). These results demonstrated that GRm@IO@PDA-aPD-L1 bioprobes could greatly increase recognition and accumulation effects in tumors. The optimum time for bioprobe accumulation at the tumor site was confirmed by fluorescence imaging; *in vivo* fluorescence imaging measurements from 0 to 96 h were performed as a contrast experiment (Fig. S14). Fluorescent dye IR-808 (200 *μ*l, 0.98 mg/ml) was coated on the GRm@IO@PDA-aPD-L1 bioprobes (200 *μ*l, 0.2 *μ*g/ml) and injected into MDA-MB-231 tumor bearing mice via the tail vein. Fluorescence imaging indicated that the largest number of bioprobes in TNBC tumors was observed at 10 h, which is consistent with the MRI results. Importantly, owing to the relatively deep laser penetration and significant SERS enhancement of the GR substrate with 785 nm illumination, SERS signals of the bioprobes in TNBC tumor-bearing mice (10 h) were directly acquired. Raman signals (1076 and 1580 cm^−1^) of 4MBA molecules collected from 10 laser points of the tumor site were stable and distinct [Fig. 4(d)]. This indicated that bioprobes with high SERS activity could be a quick and simple strategy for PD-L1 expression detection in TNBC cells. These results provide a new approach for *in vivo* PD-L1 expression detection based on the strength of the SERS-MRI dual-modal bioprobes and offers a new method to analyze PD-L1 expression in breast tumors.

## CONCLUSION

In order to effectively detect TNBC-related PD-L1 expression, GRm@IO@PDA-aPD-L1 bioprobes with SERS-MRI dual-modal imaging functions were developed. GRs and IO NPs served as the SERS and MRI platforms, respectively, to detect PD-L1 expression. SERS and MRI imaging exhibit complementary features for PD-L1 expression detection *in vitro* and *in vivo*. aPD-L1 was selected as an antibody to boost the detection specificity and improve the ability of bioprobes to target tumor sites with PD-L1 expression. PD-L1 expression in TNBC cells was accurately imaged/detected via *in vitro* SERS imaging/signal modes owing to the single-cell-level detection sensitivity of SRES; SERS signals were stable and evident. Importantly, MRI and SERS signal dual-detection modes were successfully utilized to directly and dynamically monitor bioprobe accumulation in TNBC tumor-bearing mice with high PD-L1 expression levels. SERS detection signals under 785 nm illumination were consistent with the MRI imaging results, indicating that the largest amount of PD-L1 expression-related bioprobes aggregated in TNBC tumors after 10 h. This work demonstrates that SERS-MRI dual-modal bioprobes exhibit great application potential in PD-L1 expression detection and will pave the way for designing functional bioprobes for TNBC clinical immunotherapy.

## METHODS

### Materials

Gold (III) chloride trihydrate (HAuCl_4_·3H_2_O), hexadecyltrimethylammonium bromide (CTAB), sodium borohydride (NaBH_4_), silver nitrate (AgNO_3_), sodium hydroxide (NaOH), 30% hydrogen peroxide (30% H_2_O_2_), 4-mercaptobenzoic acid (4-MBA), polyacrylic acid (PAA), iron (III) chloride hexahydrate (FeCl_3_·6H_2_O), iron (II) chloride tetrahydrate (FeCl_2_·4H_2_O), ammonium hydroxide (NH_3_·H_2_O), amine-PEG-thiol (NH_2_-PEG-SH_2_), dopamine (DA), tris (hydroxymethyl) aminomethane (tris-HCl buffer, pH = 8.5), and 1-ethyl-3-(-3-(dimethylamino) propyl) carbodiimide hydrochloride (EDC·HCl) were all obtained from Aladdin Reagent Co.Ltd. (Shanghai, China). N-Hydroxysuccinimide (NHS) was purchased from Sigma-Aldrich (St. Louis, MO, USA). Anti-PD-L1 (aPD-L1), Hoechst33342, IR-808, sevoflurane, trypsin-EDTA (0.25%), and Dulbecco's modified Eagle's medium (DMEM) were purchased from Gibco Life Technologies (Carlsbad, CA, USA). All reagents were of analytical grade and were used without further purification. Milli-Q ultrapure water was used for all the experiments.

### Synthesis of GRm (Gold nanorods with 4-mercaptobenzoic acid, 4-MBA)

Gold nanorods were prepared using the seeds growth method.^28^ For the first gold seed preparation, 20 *μ*l of 50 mM HAuCl_4_, 5 ml of 0.1 M CTAB, and 24 *μ*l of 0.1 M NaBH_4_ were mixed at a high stirring rate for few minutes to synthetize seeds. Next, the growth solution was prepared by mixing 10 ml of 0.1 M CTAB, 100 *μ*l of 50 mM HAuCl_4_, 8 *μ*l of 0.1 M AgNO_3_, and 40 *μ*l of 1 M NaOH for 5 min. Next, 56 *μ*l of H_2_O_2_ (30%) was added into the growth solution until the color of the solution faded. Finally, 30 *μ*l gold seeds was added to the resulting solution, stirred for 30 s, and kept in the dark for 8 h. Gold nanorods (GR) were obtained by centrifugation at 10 000 rpm for 10 min, three times. To prepare 4-MBA-coated gold nanorods (GRm), 5 ml of 1.25 mM GR solution was mixed with 1 ml of 10^−4^ M 4-MBA signal molecules and stirred for 30 min. The GRm products were collected by centrifugation at 10,000 rpm for 10 min three times. The final GRm products were used for the Raman test (laser wavelength: 785 nm; laser power: 26.7 mW, 25%; lens: 50 × objective).

### Synthesis of iron oxide nanoparticles (IO NPs)

Ultra-small IO NPs were prepared via a one-step water-dispersible method.^36^ 50 ml of 0.21 g/mL PAA solution was passed through N_2_ for 40 min to remove O_2_, heated to 80 °C, and then, 0.145 g FeCl_3_·6H_2_O and 0.0554 g FeCl_2_·4H_2_O were rapidly added to the PAA solution while stirring intensely. Next, 15 ml of 28% NH_3_·H_2_O was injected to adjust the pH value of the solution to 9–10. When the color of the solution turned black, mixing and heating were continued for 1 h during cooling. Using a dialysis bag of 14 000 Da, 48 h of dialysis was performed; ultra-small IO NPs with carboxyl-capped ends were obtained.

### Synthesis of PD-L1 antibody-conjugated GRm@IO (GRm@IO@PDA-aPD-L1)

A quantity of 12 mg of NH_2_-PEG-SH_2_ was added into 5 ml of a 1.25 mM GR solution and mixed for 8 h. Following this, 2 ml of 8 mg/ml EDC·HCl and 2 ml of 6 mg/ml NHS were mixed with 2 ml of a 1.25 mM IO NPs solution for 30 min with stirring to activate the –COOH of IO. Additionally, 2 ml of a 1.25 mM activated IO NP solution was added to 5 ml of a 1.25 mM GRm solution. These two treated samples were stirred at 600 rpm for 12 h, then centrifuged at 10 000 rpm for 20 min three times to wash the probes and obtain GRm@IO NPs. Under an alkaline environment, 600 *μ*l of 28% NH_3_·H_2_O, 8 ml of ethanol, 13 ml of H_2_O, and 5 ml of 0.2 *μ*g/ml GRm@IO NPs were wrapped with 2 ml of 10 mg/mL dopamine by stirring for 5 h. Then, GRm@IO@PDA NPs were isolated by centrifugation (10 000 rpm, 10 min) using Tris-HCl buffer (pH 8.5), washed three times, and redispersed in Tris-HCl of the same volume. Next, 10 *μ*l of 0.98 *μ*g/ml aPD-L1 was added into 1 ml of GRm@IO@PDA NPs, and the reaction was shaken at 600 rpm for 12 h at 25 °C in the dark. Finally, the products were centrifuged at 10 000 rpm for 10 min three times to obtain the GRm@IO@PDA-aPD-L1 bioprobes.

IR 808 marked GRm@IO@PDA-808-aPD-L1 bioprobes were conjugated during PDA modification. A quantity of 200 *μ*l of 0.98 mg/ml IR-808 were wrapped with dopamine by stirring for 5 h. Following this, GRm@IO@PDA-808 NPs were obtained by centrifugation and redispersed in Tris-HCl buffer. GRm@IO@PDA-aPD-L1–488 was synthetized using Alexa Fluor 488 marked aPD-L1 via the aforementioned method. Next, 20 *μ*l of 0.5 mg/ml Alexa-488-aPD-L1 was added to the GRm@IO@PDA solution and stirred for 12 h. GRm@IO@PDA-aPD-L1–488 probes were obtained by centrifugation.

### Cell experiments

MDA-MB-231 tumor and MCF-7 cells were purchased from the National Collection of Authenticated Cell Cultures in Shanghai, China, and cultured in DMEM medium (Gibco, Thermo Fisher Scientific, Waltham, MA, USA) supplemented with 10% fetal bovine serum (PAN-Biotech, Aidenbach, Germany) at 37 °C in 5% CO_2_ atmosphere (Heracell carbon dioxide incubator-1501, Thermo Fisher Scientific).

For cytotoxicity testing, 10^4^ of both MDA-MB-231 or MCF-7 tumor cells were cultured individually overnight in 96-well plates with 100 *μ*l of DMEM (10% FBS, 2% penicillin-streptomycin) per well. Next, GRm@IO@PDA and GRm@IO@PDA-aPD-L1 bioprobes with a range of Fe concentrations (0–60 *μ*g/ml) were added in to the above 96-well plates for another 24 h incubation. The culture medium was removed from each cell and replaced by CCK-8 solution (10 *μ*l of CCK-8 mixed with 190 *μ*l of serum-free medium per well) and incubated for 1 h. Absorbance at 450 nm was measured with a microplate reader (SpecrtaMax 190, Molecular Devices, San Jose, CA, USA). For confocal laser scanning microscopy (CLSM) test, 10^5^ of MDA-MB-231 or MCF-7 cells were cultured in 200 *μ*l of DMEM (10%FBS, 2% penicillin-streptomycin) for 12 h until the cells stick to the wall of six-hole plates, then 200 *μ*l of 0.2 *μ*g/ml GRm@IO@PDA-aPD-L1–488 was added and cells were cultured at 37 °C for 0, 8, or 24 h for fluorescence confocal experiments using a TCS SP8 multiphoton microscope (Leica, Wetzlar, Germany). For SERS imaging, 10^5^ of MDA-MB-231 or MCF-7 cells were cultured in 200 *μ*l of DMEM (10% FBS, 2% penicillin-streptomycin) for 12 h until confluency, Next, 200 *μ*l of 0.2 *μ*g/ml GRm@IO@PDA-aPD-L1 was added to each well and cultured at 37 °C for 0, 8, or 24 for SERS experiments using Labspec 6 software (Horiba Scientific, Piscataway, NJ, USA). For MR imaging, 10^5^ of both MDA-MB-231 or MCF-7 cells were incubated in six-well plates for 12 h and then 100 *μ*l, 0, 0.03, 0.12, 0.18, or 0.2 *μ*g/ml of GRm@IO@PDA-aPD-L1 was added for 8 or 24 h. 250 *μ*l of Trypsin-EDTA (0.25%) was added to digest the cells, which were subsequently washed with PBS at 1000 rpm for 5 min three times. Cells were then dispersed in PBS, and MR imaging experiments were performed using a MesoMR23-060H-I analyzer (MSITECH, Singapore).

### Animal experiments

Female ICR (London, UK) mice (7–8 weeks old) and female BALB/c-nu mice (5–6 weeks old) were purchased from Hangzhou Ziyuan Biotechnology Co. Ltd. (Hangzhou, China). All animal experiments were reviewed and approved by the Regional Ethics Committee for Animal Experiments of Ningbo University [permit no. SYXK (Zhe) 2019–0005].

The biocompatibility of bioprobes *in vivo* was evaluated in female ICR mice. Mice were divided into three groups and injected with 100 *μ*l PBS or the same volume containing bioprobes with different Fe concentrations (0.1 or 0.2 *μ*g/ml) via the tail vein. After 14 days, mice were killed after cervical dislocation under anesthesia, and blood and major organs were collected for hematological analysis and hematoxylin-eosin (H&E) section staining.

BALB/c-nu mice were subcutaneously implanted with 1 × 10^7^ MDA-MB-231 tumor cells on the front of the right leg. After two weeks, the tumor volume was increased to 100 mm^3^ for subsequent MRI and SERS detection. SERS and MRI experiments were performed on tumor-bearing mice using a LabRAM Odyssey spectrometer (Horiba Scientific) with Labspec 6 software and a MAGNETOM Vida syngo MR XA10 MRI scanner (Siemens Healthineers, Erlangen, Germany). All mice were then injected with 100 *μ*l of 0.2 *μ*g/ml GRm@IO@PDA-aPD-L1, and the accumulation was observed using MR equipment. MRI images were acquired at different time points(0, 2, 4, 10, 12, 24, and 48 h). The MRI field of view was selected according to the breast cancer type. According to the MRI results, the SERS spectra of *in vivo* tumors were acquired 10 h after tail vein injection using LabRAM microscopy.

The biodistribution of bioprobes was observed using fluorescence imaging equipment (IVIS spectroscopy, PerkinElmer, Waltham, MA, USA). Tumor-bearing mice were injected with 100 *μ*l of 0.2 *μ*g/ml GRm@IO@PDA-808-aPD-L1 bioprobes and IR 808 via tail vein, followed by real-time monitoring. After IR-808 labeling, GRm@IO@PDA-808-aPD-L1 exhibited fluorescence with excitation at 740 nm and emission at 790 nm.

### Statistical analysis

All data were presented as mean ± standard deviation for at least three independent experiments. The measurement data were analyzed statistically using Excel.

## SUPPLEMENTARY MATERIAL

See the supplementary material for the following details: TEM images of GRm with different aspect ratios and spectra of GRm with different aspect ratios (Fig. S1); zeta potential of the GRm, GRm@IO, GRm@IO@PDA, and GRm@IO@PDA-aPD-L1 (Fig. S2); HRTEM images of GRm@IO@PDA (Fig. S3); HADDF and elemental mapping images for N, Au, and Fe in GRm@IO@PDA (Fig. S4); particle sizes of GRm, GRm@IO, GRm@IO@PDA, and GRm@IO@PDA-aPD-L1 (Fig. S5); CLSM images of GRm@IO@PDA-aPD-L1–488 (Fig. S6); cell viabilities of MCF-7 cells incubated with bioprobes (Fig. S7); CLSM images of MCF-7 cells with Alexa Fluor 488 marked bioprobes (Fig. S8); flowcytometry results of the expression level of PD-L1 in MDA-MB-231cells and MCF-7 cells, and GRm@IO@PDA-aPD-L1–488 bioprobes incubated with MDA-MB-231cells and MCF-7 cells for 24 h (Fig. S9); SERS mapping and MRI images of MCF-7 cells incubated with GRm@IO@PDA-aPD-L1 (Fig. S10); routine analysis of the WBC, RBC, HCT, MCV, HGB, PLT, MCH, and MCHC after injecting bioprobes (Fig. S11); HE results from the lung, liver, spleen, kidney, and heart of ICR white nude mice after injecting the bioprobes (Fig. S12); MRI images of MDA-MB-231 cells bearing mice injected GRm@IO@PDA at different time points (Fig. S13); and fluorescence imaging of MDA-MB-231 tumor in BALB/c female nude mouse at different timepoints (Fig. S14).

## Data Availability

The data that support the findings of this study are available within the article and its supplementary material.
